# Aspirin and Age Related Macular Degeneration; the Possible Relationship

**Published:** 2013

**Authors:** Yan Wu, Wei Zhu, Yan-Hong Li, Jing Yu

**Affiliations:** 1 Department of Ophthalmology, Affiliated Tenth People’s Hospital of Tongji University, Shanghai 200072, China,; 2 Department of First Clinical Medical College, Nanjing Medical University, Nanjing, Jiangsu, China

**Keywords:** Aspirin, Age Related Macular Degeneration, Neovascularization

## Abstract

Age-related macular degeneration (AMD) is becoming the leading cause of blindness in developed countries. The exact etiology and pathophysiology of AMD is still unclear. A number of risk factors of AMD have been recognized, such as cigarette smoking, a family history of AMD and being Caucasian. On the other hand, aspirin is a widespread medication, which is thought to be associated with the prevalence or the survival of myocardial infarction and cancers. However, the evidence from the epidemiological studies has been contradictory and no persuasive conclusions have been made. Several problems, such as the parameters of aspirin use, the inclusion and exclusion of the participants and the required long-term follow-up, made it hard to conclude a definite relationship between aspirin use and AMD. Aspirin, as an anti-inflammatory agent, could prevent the inflammation and decrease the inflammatory damage, and might act as a deterrent for the progression of AMD. However, aspirin is an anticoagulant which might increase the risk of ocular hemorrhage in AMD patients. Decades ago, the use of aspirin was reported associated with decreased rates of CNV among AMD patients nevertheless recently, the association between aspirin use and increased risk of neovascular AMD was identified. Therefore, these current results should be challenged and acknowledged by well-designed, large-scale and long term follow-up studies. A consultation might be needed when aspirin is used in the neovascular AMD patients.

## Introduction

Age-related macular degeneration (AMD), which was first introduced in 1875 in medical literature, is now regarded as one of the most common cases of blindness [1]. The prevalence of AMD increased exponentially each decade after fifth decades of age [2] and in the developed areas, AMD was the cause of more than half of the blind in the elderly [3]. Furthermore, AMD is a progressive disease and multifactorial condition in terms of etiology. Other than age and genetic predisposition, several epidemiologic risk factors such as cigarette smoking, family history of AMD and Caucasian race were also reported [4, 5]. Besides, several systemic disorders including hypertension, cardiovascular disease, stroke, and diabetes mellitus were associated with the incidence of AMD as reported by several studies [6-9]. However, the associations between systemic diseases with AMD were inconsistent in each study. It should be noted that more research is required to get further evidence on these theories and pathophysiology of the disease.

The etiology and pathophysiology of AMD remain unclear. In general, AMD is a multifactorial disease in which oxidative damage, inflammatory reaction and abnormal immune response are involved [8, 10, 11]. The understanding of the pathophysiology of AMD has improved through several studies however the attributes of each pathological causal agent on the progress of AMD is largely unknown. It is because of the complexity of the etiology and pathology of AMD that effective therapy and prophylaxis have yet to be developed. Anti-VEGF therapy is regarded as effective for the wet AMD patients, however, it is still not a curative therapy [12]. Further research on the detection of the core pathophysiology of AMD is required.

Clinical researches are detecting the association between certain factors and AMD. The results of clinical research, in turn, would lead to advanced understanding of the pathology and potential improved therapy. Aspirin is a widespread medication which is associated with the prevalence or the survival of myocardial infarction and cancers [13-15]. The association between aspirin use and risk of AMD was reported in several cross-sectional studies, while no accordant conclusion was obtained [16, 17]. We conducted a meta-analysis through pooling the relevant studies together and no significant results were detected [18]. However, taking the inherent limitations of that meta-analysis into account, the conclusion should be considered with caution. Recently, several well-designed large-sample studies reported the association between aspirin use and risk of AMD and demonstrated valuable and interesting results [19-21]. The purpose of this manuscript is to review the association between aspirin use and the risk of AMD. 

**Classification and Clinical symptoms of AMD**

Two hallmarks, presence of drusen and choroidal neovascularization (CNV), are of great importance in the diagnoses and classification of AMD. Drusen is usually the first detected clinical finding in AMD and the presence of CNV is a sign of an aggravated AMD state [22, 23]. According to the former studies, drusen are classified by the diameter as small (<63 μm), medium (63 to 124 μm) and large (>124 μm). Several clinical classifications exist nowadays and the most often used is the classification proposed by the Age-Related Eye Disease Study (AREDS, supported by the National Institutes of Health) [24]. A total of five categories of AMD are defined: asymptomatic AMD; early AMD; intermediated AMD; advanced non-neovascular AMD and advanced neovascular AMD. In the early AMD, only a few medium-size drusen presented while in the intermediate AMD, numerous medium-size and at least one large drusen presented. However, the center of the macular hasn’t been involved. In the advanced non-neovascular AMD, the center of macular is involved. The presence of CNV and its complications demonstrates the advanced neovascular AMD. 

In the early stage of AMD, the damage of visual acuity and visual function is not apparent and quite a few patients are diagnosed from routine examinations. However, the presence of advanced AMD, especially the neovascular AMD, is a significant threat to the vision. Appropriate measures should be conducted in all the different stages [25].

**Pathophysiology of AMD**

As defined beforehand, the pathophysiology of AMD is still unclear. AMD is a progressive disease, which affects retinal pigment epithelium (RPE), photoreceptors, Bruch’s membrane and choriocapillaris [1]. Several pathologic processes, including oxidative damage, inflammatory reaction and abnormal immune response, are regarded as core in the progression of AMD.

Oxidative response, which plays a key role in numerous diseases, works in the development and progression of AMD. The epidemiological research demonstrated that a reduced exposure to an oxidative environment and the additional supplementation of antioxidant diet/drug would reduce the prevalence and progression of AMD [26-28]. The visual acuity impairment often occurs when photoreceptor, RPE, and Bruch’s membrane are damaged by the oxidative stress [29]. More research has reported the extended effects of oxidative response on AMD which provided novel theories on the pathophysiology of AMD [30]. 

It is widely accepted that inflammation plays an important role in the development and progression of AMD [31, 32]. Some inflammatory markers, such as C-reactive protein, intercellular adhesion molecule, interleukin-6, are abnormally expressed in the AMD cases [33-36]. Furthermore, some inflammatory cells, including macrophages and microglia, were involved in the AMD [37, 38]. Considering that AMD is not a classical inflammatory disease, more studies required to be focused on the effects of parainflammation in the AMD [39]. Parainflammation is defined as an adaptive response to stress, such as aging, oxidative stress and oxidized lipoproteins [40]. It is a condition between normality and acute inflammation, which functions as a path to maintain the normal retina and restore the damaged tissues [41]. In general, it is regarded as a beneficial effect. However, it should also be noted that the abnormal parainflammation is probably a cause of the onset or progression of AMD [42, 43]. Several studies had aimed to treat AMD by targeting the inflammatory pathway. IL-10 regulates macrophage activity, which shows the potential to be a therapeutic target. An IL-10 knocked-down mouse is used as an animal model of CNV caused by AMD. The anti-inflammatory intervention revealed improvement of the CNV [44]. Recombinant TSG-6, as an anti-inflammatory protein, showed the effect of treating AMD through an intravitreous injection into the animal model [45]. However, it is a long way to develop a definitive and substantial treatment of AMD by focusing on the inflammatory reactions.

Although the eye is an “immune-privileged” site, AMD is regarded as immune mediated disease as well [46]. Various evidences exist about the role of immune reaction on the AMD. Histological study of drusen and CNV proved that immunologic dysfunction also existed in humans [47]. The complement system, which is part of immune system, is thought to play an important role in the development of AMD [48]. Furthermore, several other immune components, such as antigen presenting cells, also contribute to AMD [49]. Nowadays, AMD is considered to be a systemic immunologic disease with local expression. Thus therapeutic strategies should be changed. In the future, advanced research on the trigger mechanisms would beneficial to develop more effective therapies for AMD.

**General Mechanism of Aspirin Action**

Aspirin is a classical non-steroidal anti-inflammatory drug (NSAIDs), which works through both cyclooxygenase (COX)-dependent pathways and COX-independent pathways. In the COX-dependent pathway, aspirin inhibits both COX-1 and COX-2 irreversibly. Cox converts arachidonic acid to prostaglandin H2 and the inhibition of COX will lead to a decreased expression of prostanoids including prostaglandin (PG)D2, PGF2α and PGE2, thromboxane A (TXA2) and prostacyclin (PGI2) [50]. Prostanoids demonstrate a wide range of biological functions including cell proliferation and migration, angiogenesis, apoptosis, and inflammatory response [51-53]. Thus, it could be concluded that aspirin played a role in these aspects.

Aspirin can also function through COX-independent pathways. The most accepted pathway is the inhibition of transcription factor nuclear factor κb (NFκB) [54]. Besides, other mechanisms of the actions of aspirin were also reported such as induction of apoptosis, DNA stabilization, TNF-α and TGF-β expression, induction of heme oxygenase-1 (HO-1) and Wnt signaling [55-58].

**Aspirin and AMD — Evidence from Experimental Research**

AMD is a multifactorial disease consisting of various kinds of pathologic processes. Aspirin is a drug with various pharmacological effects. It is logical to conclude that aspirin could produce some effects on the AMD. However, the direct evidence about the relationship between aspirin use and the development of AMD are unproven. We can only presume the possible effects of aspirin on AMD from the current experimental research. 

Firstly, aspirin might generate some beneficial effects by delaying the progression of AMD. Inflammation is a core physiopathological factor in AMD. The proinflammatory genes such as COX-2, MCP-1 and IL-8 are up-regulated in AMD [59]. Aspirin, which is an anti-inflammatory agent, could prevent the inflammation and decrease the inflammatory damage. Considering that aspirin might affect through the COX-independent pathway, more specific results are expected. NFκB is a key target of aspirin in the COX-independent pathway [60, 61]. Other target sites of aspirin, such as Wnt and HO-1 pathways, were also reported to be related with AMD [62, 63]; they are the possible linkages between aspirin and AMD. However, all the presumptions above are based independent experiments. More relevant, directed and well-designed experiments are required as proof. Therefore, the opinion that aspirin is a protectant for AMD should be considered with caution.

However, harmful effects might be produced by aspirin on AMD as well. A most recent study revealed that a NSAID, which is a meclofenamic acid, would block the gap junction communication between the RPE cells. This phenomenon was also observed in other NSAIDs, including aspirin [64]. It presumed that the damaged retinal microenvironment would worsen the progression of AMD. Considering that aspirin has a widespread use as an anticoagulant, the risk of ocular hemorrhage is predicted to increase in AMD patients, especially in the neovascular AMD patients. Also, the parainflammation in AMD might interfere with restoration and reconstruction. Considering no powerful evidence of the definitive effects of aspirin on AMD, the beneficial or harmful effects of aspirin on the AMD have not been reasonably evaluated. Advanced relevant studies are required to further explore aspirin use and its likely effects.

## ASPIRIN AND RISK OF AMD

**As a protective factor**

A retrospective consecutive case series study was also conducted to investigate the relationship between the use of aspirin or stains and the risk of CNV in the AMD patients [65] (Table 1). The use of aspirin or statins was reported associated with decreased rates of CNV among AMD patients in the case series study. 

The Physician’s Health Study (PHS) [66] is a randomized-controlled trial (RCT) designed for the effects of aspirin and beta carotene in the prevention of cardiovascular disease and cancer. A total of 22,071 US participants were enrolled in the baseline and a minimum of 7 year follow-up was conducted. The results of PHS tend to exclude beneficial effects on the treatment of AMD. However, in the hypertension group, aspirin use might reduce the incidence of AMD ([relative risk] RR: 0.35; 95% CI: 0.15 to 0.83) after other risk factors have been adjusted. The hypertension group also revealed mild protective effects compared with the non-hypertension group. This finding provides the rationale for the advanced research about the association between aspirin, cardiovascular disease and AMD.

**As a risk factor**

The European Eye Study, which is a population-based cross-sectional study, focused on the relationship between aspirin use and incidence of AMD [67]. A total of 4691 participants of 65 years or older from 7 centers in northern to southern Europe were enrolled in the study. The results demonstrated that frequent aspirin use was associated with both early AMD and wet late AMD, and the risk rose with increasing frequency of consumption. The Singapore Indian Eye Study, a study enrolling 3207 ethnic Indians aged 40 years or older, also reported an increased risk of early AMD in the aspirin users [19]. Following adjustment for age, smoking and previous cataract surgery, the odds ratio (OR) was 1.50 (95% CI: 1.01 to 2.22); however, no significant association was detected when adjusting for cardiovascular disease (OR: 1.38; 95% CI: 0.89 to 2.14). This interesting result showed the aspirin use might be part of the risk of AMD, however the subjects with a cardiovascular disease history should be considered with more targeted study.

There were also the two most recent studies reported by Klein et al [21] and Liew [20]. Klein et al reported the data from 10 years of follow-up of “The Beaver Dam Eye Study”. The association between the use of aspirin and the incidence rate of early, late and two subtypes late AMD were detected. A small but statistically significant increase in the late AMD incidence was identified (hazard ratio [HR], 1.63; 95% CI: 1.01-2.63). When considering the two subtypes of late AMD, the neovascular AMD and pure geographic atrophy, the use of aspirin was associated with the incidence of neovascular AMD (HR: 2.20; 95% CI: 1.20-4.15). However, the results of the fivr year follow-up of Beaver Dam Eye Study demonstrated aspirin was associated with neither early nor late AMD [68]. Comparing the results of different follow-up durations in the same clinical trial, a possible explanation is that the effect of aspirin on the incidence of AMD is quite weak and only a factor in the long term (maybe 10 years) for an indicator of a significant harmful effect. As the first study reported, regarding the increased rate of AMD in the aspirin users, this study was also questioned by the definition of the aspirin use and the potential bias in the study structure [69]. Apart from the existing limitations, the conclusions of this study would provide us with viable data. Another study, which was the Australian population-based cohort with a 15 year follow-up, reported a similar result as Klein’s study [20]. The association between aspirin use and risk of neovascular AMD (OR: 2.46; 95% CI: 1.25-4.83) was detected but not with the geographic atrophy (OR: 0.99; 95% CI: 0.59-1.65). The relevant confounding factors, such as age, gender, smoking, history of cardiovascular disease, were adjusted and it concluded that regular aspirin use is an independent risk of AMD.

**As an unrelated factor**

A cross-sectional study in Korea was conducted to explore the potential risk factors and aspirin was reported to be not associated with the risk of AMD [70]. The Blue Mountain Eye Study is population-based cohort study about the visual and common eye diseases in the urban population aged over 49 years [71]. A total of 3654 participants were in the baseline and a 5 year follow-up was conducted. The use of aspirin was not associated with the incidence of AMD, in neither the baseline (OR: 0.9; 95% CI: 0.4 to 0.9) nor the follow-up (OR: 1.3; 95% CI: 0.8 to 2.0). In the different frequency aspirin intake groups (less than once per week and once per week or more), the OR values were not statistical different. In a matched-pair case-control study, the epidemiological risks of neovascular AMD were detected [72]. The extremely discordant sib-pair study design made the conclusions persuasive while the use of aspirin was not associated with the risk of AMD according to the results. However, this study did not provide more detailed data about the use of aspirin and advanced discussions were limited. The Age-Related Eye Disease Study (AREDS) is a study of great importance in the study of AMD [73]. The use of aspirin was also mentioned in the reports of the AREDS and no positive result was detected. It would be significant to analyze this part of the data in advanced studies since interesting results might be attained. A matched case-control with 18,007 participants enrolled was conducted to explore the relationships of different frequently used drugs and incidence rate of AMD [74]. This large sample data was from the General Practice Research Database (GPRD) of the United Kingdom and no association was detected in this study as well.

The Women’s Health Study (WHS) [75] is a RCT about the effects of the adoption of 100 mg of aspirin on alternate days. In this RCT, 39,876 females aged 45 years or older were enrolled and over 10 years of treatment and follow-up were conducted. The outcome was that AMD was responsible for a reduction in best-corrected visual acuity to 20/30 or less. This study was different from the preceding studies. The results of this large-scale study showed that the use of aspirin is neither beneficial nor harmful to the development of AMD. Although RCT design could discharge the potential bias and confounding factors, credible conclusions could be reached.

Meta-analysis is an effective way to pool the existing consubstantial studies together and provide more powerful evidence. We have conducted a meta-analysis about the use of aspirin and risk of AMD [18]. Through pooling 10 existing studies (2 RCTs, 4 case-control studies and 4 cohort studies), we concluded that aspirin use provided neither beneficial nor harmful effects on AMD. However, this conclusion should be considered with reasonable doubt because of the various studies’ limitations.

**Aspirin and ocular hemorrhage in AMD**

As an anticoagulant, aspirin was reported associated with the increased risk of hemorrhage, especially the gastrointestinal area [76]. Aspirin was regarded as a risk factor of ocular hemorrhage [17, 77]. A report of 15 cases of ocular hemorrhage in the AMD demonstrated a potential risk of aspirin use [77]. Another study demonstrated that aspirin used in the neovascular AMD would raise the risk of massive intraocular hemorrhage. It also documented that aspirin should be prescribed only for absolute systemic indications [78]. However, this opinion was challenged by other studies, in which the association between aspirin use and risk of ocular hemorrhage was not detected [16, 79]. A retrospective case series of 520 patients about the safety of the intravitreal anti-VEGF agent therapy, aspirin was found not to be a risk of ocular hemorrhage [79]. However, no accordant conclusion has been universally reached. More advanced studies in the future will provide us with a better understanding of the overall etiology.

**Table 1:** Summary of the evidence from the main clinical studies

Effect	Author, Year	Study	Number of cases	Study design	Site	Follow-up	Results RR/OR/HR (95% CI)
Protective factor	Wilson HL, 2004 ^65^	None	326	Case-series	Americas	None	HR: 0.63 (0.40-0.98)
	Christen WG, 2001 ^66^	Physician’s Health Study	22,071	RCT	Americas	> 7 years	RR: total: 0.78 (0.46-1.32) no hypertension: 0.95 (0.63-1.44) hypertension: 0.35 (0.15-0.83)
Risk factor	de Jong PT, 2012 ^67^	European Eye Study	4,691	Cross-sectional	Europe	None	OR: monthly: 1.02 (0.47-2.22) weekly~daily: 1.25 (0.53-2.96) daily: 0.95 (0.61-1.46)
	Cheung N, 2013 ^19^	Singapore Indian Eye Srudy	3,207	Cross-sectional	Asia	None	OR: CVD not adjusted: 1.50 (1.01-2.22) CVD adjusted: 1.38 (0.89-2.14)
	Klein BE, 2012 ^21^	Beaver Dam Eye Study	4,926	Cohort	Americas	> 10 years	HR: late AMD: 1.63 (1.01-2.63) neovascular AMD: 2.20 (1.20-4.15) pure geographic atrophy: 0.66 (0.25-1.95)
	Liew G, 2013 ^20^	None	2,389	Cohort	Australia	15 years	OR: neovascular AMD: 2.46 (1.25-4.83) geographic atrophy: 0.99 (0.59-1.65)
Unrelated factor	Moon BG,, 2012 ^70^	None	10,449	Cross-sectional	Asia	None	OR: 1.22 (0.86-1.73)
	Wang JJ, 2003 ^71^	Blue Mountains Eye Study	3,654	Cohort	Australia	5 years	OR: baseline: 0.9 (0.4-0.9) follow-up: 1.3 (0.8 to 2.0)
	DeAngelis MM, 2004 ^72^	None	146	Case-control	Americas	None	OR: 1.37 (0.57-3.32)
	Clemons TE, 2005 ^73^	Age-Related Eye Disease Study	4,425	Cohort	Americas	6.3 years	OR: bilateral drusen (neovascular) 1.30 bilateral drusen (central GA) 1.00 advanced AMD (neovascular) 1.12 advanced AMD (central GA) 0.25
	Douglas IJ, 2006 ^74^	General Practice Research Database	18,007	Case-control	Europe	None	OR: crude: 1.22 (1.17–1.27) adjusted: 1.00 (0.96–1.04)
Christen WG, 2009 ^75^	Women’s Health Study	39,876	RCT	Americas	10 years	HR: 0.82 (0.64−1.06)

RR: relative risk; OR: odds ratio; HR: hazard ratio; GA: geographic atrophy; CVD: cardiovascular disease; RCT: randomized-controlled trials.

## CONCLUSION

The relationship between aspirin and risk of AMD has long been suggested and it drew more attention following association between aspirin use and risk of AMD was reported. The experimental evidence, as seen today, is extremely limited and the presumptions that have been made are not based on persuasive empirical data. Future experiments might provide novel perspective on this issue. The evidence from clinical studies is abundant; however, no unified conclusion has been reached. Several problems, such as the definition of aspirin use, the inclusion and exclusion of the participants and the required long-term follow-up, made it hard to conclude the confirmatory relationship between aspirin use and AMD. It also should be noted that aspirin use might be related with increased risk of ocular hemorrhage in the AMD patients, while this association is still under investigation. These existing results should be challenged and acknowledged by more well-designed, large-scale and long-term follow-up studies. As a widely used drug, aspirin is important in the treatment of several systemic diseases. Aspirin use in the AMD patients should be based on a consultation.

## DISCLOSURE

Conflicts of Interest: None declared.
